# Resonance-enhanced multiphoton ionization time-of-flight mass spectrometry for the real-time analysis of a retronasal aroma compound from a cumin sandwich

**DOI:** 10.1007/s44211-026-00907-z

**Published:** 2026-04-03

**Authors:** Hazuki Uno, Masaaki Ukita, Tomohiro Uchimura

**Affiliations:** Department of Materials Science and Engineering, Graduate School of Engineering, University of Fukui, 3-9-1 Bunkyo, Fukui, 910-8507 Japan

**Keywords:** Retronasal aroma, REMPI-TOFMS, Real-time measurement, *p-*Cymene

## Abstract

This study was focused on developing a process to objectively determine the release behavior of retronasal aromas in food in real time, which could contribute to the development of foods better suited to consumer preferences. In food, organic compounds with low molecular weight easily vaporize to elicit a retronasal aroma, and such compounds are referred to as volatile. In this study, the release of aroma during the eating of small sandwiches was measured and verified in real-time via resonance-enhanced multiphoton ionization time-of-flight mass spectrometry (REMPI-TOFMS). Cumin and small slices of bread were used as food samples that would easily vaporize and could be detected in the breath of a person eating them. Laser pulses emitting at 266 nm during REMPI selectively detected the naturally occurring organic compound *p*-cymene in cumin. While it was being chewed, the time profile for *p*-cymene as a retronasal aroma compound showed the increases and decreases in the detection of this food in breaths released in a 6 s cycle (3 s intake, 3 s exhalation). Overall, the retronasal aroma increased and then decreased. The total ion signals of *p*-cymene obtained during chewing (integrated area under the time profiles) were slightly smaller when eating 1 g of bread than when eating 3 or 7 g. The bread samples were mixed with saliva during chewing, and the oral cavity became relatively saliva-rich. In other words, the bread was easily converted to a liquid food, and the vaporization of the *p*-cymene in cumin seemed to be suppressed with smaller slices of bread. Moreover, a fit function for the obtained time profile was proposed in the present study. The profile was constructed by combining the subtracted values of the two logistic functions and a trigonometric function. As a fit result, the start and end times of the overall transient time profile were both earlier when eating 1 g of bread than when eating 3 or 7 g; the start time was numerically ca. 3 s earlier, and on average, *p*-cymene was found to be emitted from the nose half a breath earlier under the present eating conditions.

**Graphical abstract:**

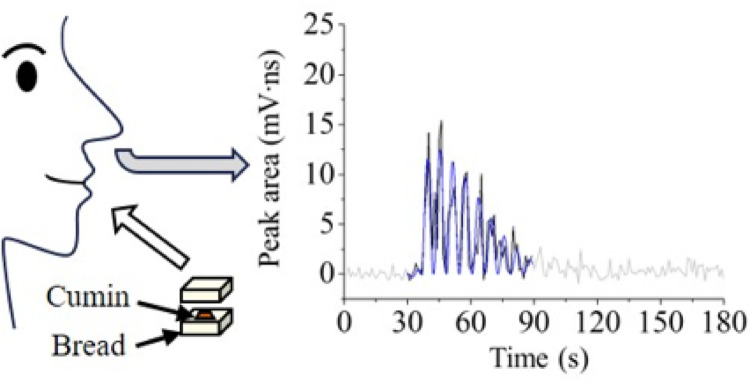

**Supplementary Information:**

The online version contains supplementary material available at 10.1007/s44211-026-00907-z.

## Introduction

In humans, aroma is perceived via two pathways: the orthonasal pathway, in which outside air enters the nose, and the retronasal pathway, in which air enters from the pharynx to the nose. Retronasal aroma is perceived as a flavor during eating/drinking and is directly connected to perceptions of the deliciousness of food [1]. Objectively evaluating the release behavior of retronasal aroma in real time could contribute to the development of foods better suited to consumer preferences. Although natural methods for sensory evaluation are conducted as simple methods, these are subjective and strongly reflect individual variability.

Instrumental analysis for the evaluation of aroma components offers data that are based on objective measurement. Gas chromatography/mass spectrometry (GC/MS) is a powerful tool for the analysis of aroma components in foods. Wang et al. identified a total of 43 volatile compounds in 17 cumin samples from different regions that were identified by headspace solid-phase microextraction (HS-SPME) followed by GC/MS [2]. Sonmezdag et al. measured the volatile compounds of biscuits via HS-SPME-GC/MS, and reported that the number of volatile compounds increased during mastication [3]. However, GC is a separation method for mixtures and it is inherently difficult to use as a tool for real-time analysis. The changes in retronasal aromas occur not only by breath but also by factors such as the situation of chewing and the volume of saliva. Therefore, an analytical method that could offer real-time measurement is highly desired.

Mass spectrometry, without GC, is one of the analytical methods for the real-time measurement of gas analysis, which includes breath gas [4, 5]. Hodgson et al. reported the retronasal aroma components of drink via atmospheric pressure chemical ionization-mass spectrometry (APCI-MS); volatile compounds could be detected in a few breaths after swallowing [6]. Szymańska et al. calculated 10 parameters from the release behavior measured using proton-transfer reaction mass spectrometry (PTR-MS), and discussed the automated data preprocessing procedure and the multivariate assessment of the panel performance [7]. However, when using the above ionization methods, not only the retronasal aroma components but also other components in the breath and atmosphere are comprehensively ionized and detected. Although this characteristic is useful for the simultaneous detection of multiple aroma compounds, it also poses a potential risk of interference among the peaks of the obtained mass spectra. Although Weber et al. introduced a novel detector comprising a separation column followed by a highly sensitive chemoresistive sensor for breath limonene, which is a reliable biomarker for liver disease [8], selective measurement using a chemical sensor is limited. For this reason, the use of highly selective MS would be useful for the real-time analysis of retronasal aroma compounds.

Resonance-enhanced multiphoton ionization time-of-flight mass spectrometry (REMPI-TOFMS) using an ultraviolet laser as an ionization source has superior selectivity, which is quite useful for the detection of analyte species from a mixture sample containing several volatile compounds [9–13]. As an example of food analysis, Zimmermann et al. reported the application of REMPI-TOFMS for the real-time analysis of volatile compounds emitted during coffee roasting [14]. In a previous study, we applied this method to the real-time measurement of flavor volatiles emitted during the cooking of rice [15]. Although a large amount of water vapor is generated during the cooking of rice, water molecules are not ionized by REMPI with nanosecond ultraviolet laser pulses, and in that study no negative influence arising from water molecules occurred. Therefore, a real-time analysis of volatile compounds in exhaled breath should be possible, because breath contains much less water vapor than the gases released when rice is being cooked. In a preliminary study, a retronasal aroma compound released during the chewing of gum was measured in real time, and the obtained transient signal was fitted by combining a function of an exponential decay curve and a trigonometric function [16]. The behavior of retronasal aroma compounds is greatly influenced by factors such as the form and quantity of food, as well as by individual differences. Therefore, quantitative analysis using a fit equation would be beneficial for the field of food development.

In the present study, we used REMPI-TOFMS to avoid interferences while evaluating the release behavior of a specific compound in retronasal aroma during eating. A cumin sandwich was used experimentally because the vaporization of compounds in cumin was considered to be minimized before eating (during sample preparation). The components released when eating different amounts of cumin and bread in a sandwich were measured in real-time, and the durations of release were evaluated and quantified using a fit function.

## Experimental

### Reagent and sample preparation

Powdered cumin (S&B Foods Inc.) was purchased from a supermarket and stored in a freezer once opened. White bread was purchased from a bakery and stored at room temperature for use within 3 days. The *p*-cymene (standard content: 98% + , FUJIFILM Wako Pure Chemical Corp.) was used to identify a component released from cumin.

A schematic of food samples and eating conditions appears in Fig. S1. Bread was cut into pieces with sides measuring either 1.5 or 3 cm in thicknesses of either 1 or 2 cm. The bread slices provided total amounts of 1, 3, and 7 g when 0.1 g of cumin was sandwiched between them for panelists to eat and swallow. As a control experiment, cumin without bread, i.e., 0 g of bread, was also eaten and swallowed. Furthermore, as a preliminary experiment, the same weight of cumin on a spoon was held in the mouth where it was neither chewed nor swallowed. Hereafter, the data are labeled as follows: S refers to the use of a spoon, the numbers 0, 1, 3, and 7 refer to the total weight in grams of each two pieces of bread, and A-D identifies each panelist by their initials in a hyphenated construction (e.g., S-A and 1-B).

### Eating conditions

Retronasal aroma gases were measured with the informed consent of four panelists chosen from our laboratory (ages 21–24, one male and three females, all nonsmokers). Panelists were instructed to avoid strong-smelling foods, such as curry or coffee, and to complete their meals at least 30 min prior to the measurement. The panelists breathed in 6 s cycles (3 s intake and 3 s exhalation) with their mouths closed under instruction. First, as a blank measurement, breath gas without a sample in the mouth was measured for 30 s, and then a sample was placed into the oral cavity. The sample of cumin on a spoon was held in the oral cavity while the lips were kept closed and without swallowing. Samples of only cumin or of cumin sandwiched between pieces of bread were placed into the oral cavity and, while the lips were kept closed, were chewed for 60 s and then swallowed. The speed of chewing was not specified; the panelists were asked to chew at their usual pace. All data were recorded for 3 min. After each measurement, each panelist rinsed the oral cavity with tap water and waited for a sufficient amount of time (at least 30 min) before the next measurement.

### REMPI-TOFMS

**Fig. 1:**
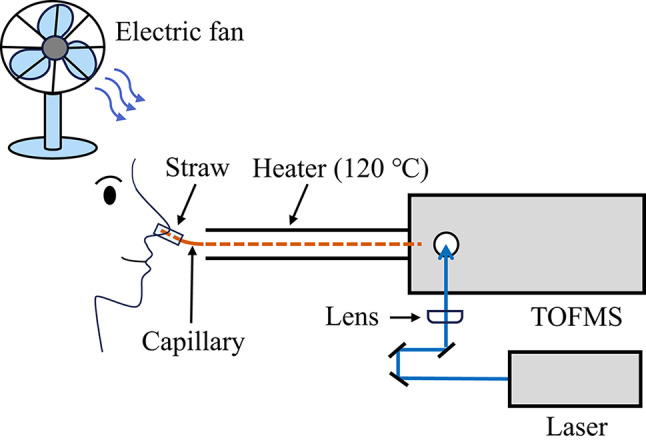
Schematic diagram for measuring retronasal aroma via REMPI-TOFMS

The experimental setup used in the present study appears in Fig. 1. A deactivated fused-silica capillary (inner diameter of 100 µm, length of 1 m, GL Sciences Inc.) was used for the sample introduction for TOFMS. For the introduction of the retronasal aroma gas to the capillary, a plastic straw (inner diameter of 1.2 cm, length of 5 cm) was taped to the capillary, and the straw then could be gently inserted into the nose of each panelist, which translates to the experiment being conducted under open-system conditions. The capillary was heated at 120 °C in order not to form water droplets inside the capillary column from exhaled breath, which could have hindered the sample introduction. Moreover, wind was blown by an electric fan beside the panelist to disperse the components from exhaled breaths to measure directly from the nose only each breath exhaled. In addition, the *p*-cymene gas generated by a standard gas generator (Permeater, PD-1C, GASTEC Corp.) was measured to confirm the reproducibility of the signal and the limit of detection (LOD).

The process for REMPI-TOFMS used in the present study has been reported elsewhere, and will be briefly described here [15]. The fourth harmonic emission of a pulsed Nd:YAG laser (GAIA II, 266 nm, 4 ns, 10 Hz, Rayture Systems Co., Ltd.) was used for REMPI. The pulse energy was set to 50 µJ, and was focused using a plano-convex lens with a focal length of 200 mm. A digitizer (AP240, bandwidth 1 GHz, sampling rate 1 GS/s, Acqiris/Agilent Technologies) was used to record the data. A time profile of the peak area for *p*-cymene was constructed by extracting the area of the molecular ion peak (*m*/*z* 134) obtained from the series of obtained mass spectra. The fitting of a time profile for retronasal aroma compounds during chewing of the cumin sandwich was performed using scientific data-analysis software (Origin 9.1, LightStone Corp.). Significant differences among the different amounts of bread were determined via one-way ANOVA statistical analysis. A Tukey’s test that employed the same software was used to discriminate among the means of the variables. Differences with a *p* < 0.05 were considered significant.

## Results and discussion

### Time profile of a retronasal aroma component when eating only cumin and when eating a cumin sandwich

REMPI-TOFMS has a high level of selectivity so that the ionizable and detectable components of cumin were the first to be confirmed. As a result, *p*-cymene was selectively detected, which was identified by GC/MS (data not shown). Moreover, the *p*-cymene gas generated by a standard gas generator (0.108–3.94 ppm) was measured via REMPI-TOFMS (n = 3). As a result, the concentration of *p*-cymene and the peak area showed good linearity (coefficient of determination (*R*^2^) of 0.9998), and the LOD was 0.098 ppm, which was obtained from 3σ of the quantified values for 0.108 ppm (data not shown).

**Fig. 2:**
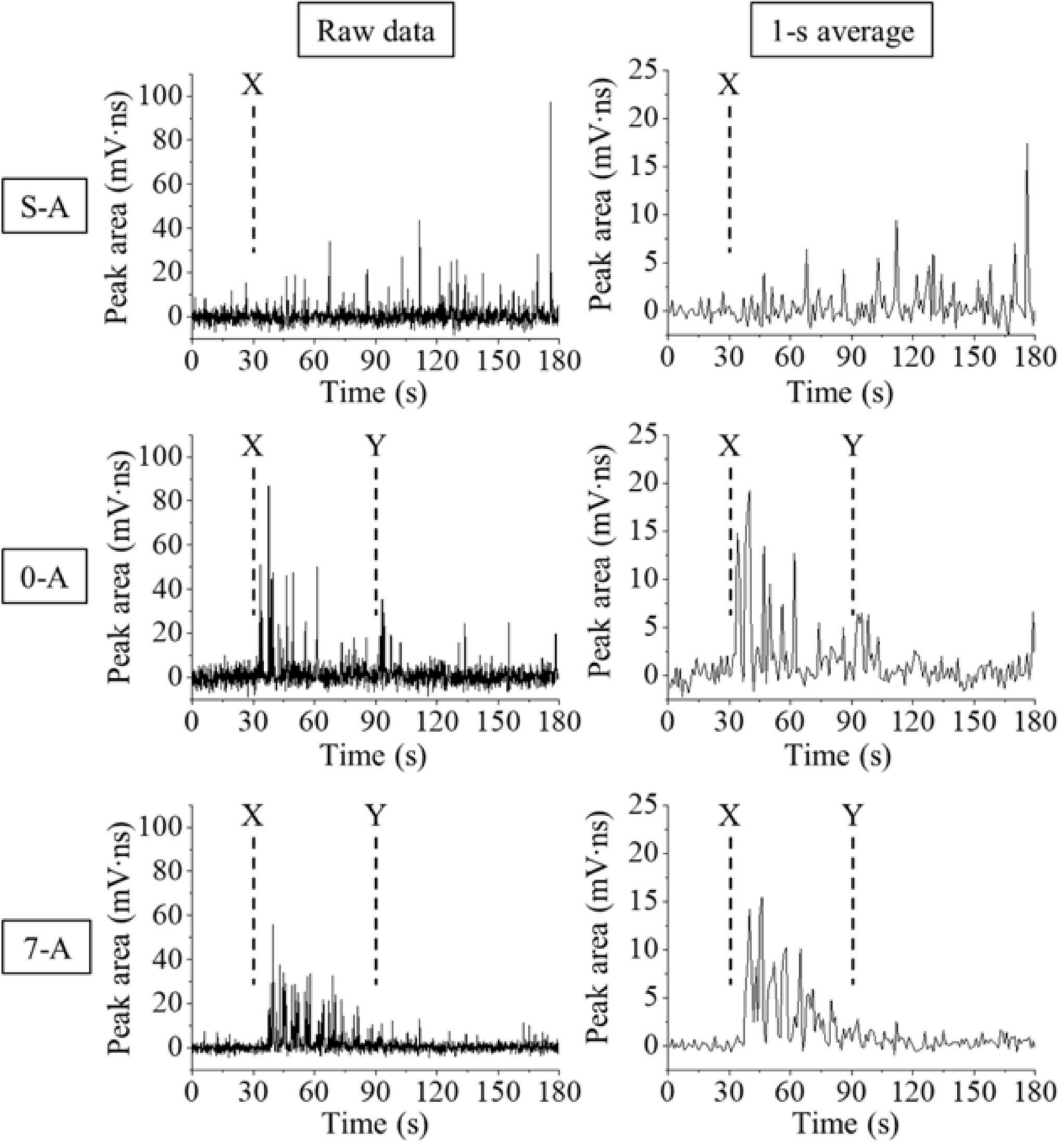
Time profiles of *p*-cymene as a retronasal aroma compound. S or the numbers 0 or 7 indicate the experimental conditions either where a spoon was placed in a mouth or the total amount (g) of two pieces of bread (0 means that only cumin was placed on the tongue). Panelist: A. (Left) Raw time profiles (for every 0.1 s), (Right) averaged time profiles for every 1 s. X indicates when the sample was put into the oral cavity (30 s after starting the recording). Y indicates when the panelist swallowed the sample (60 s after the start of eating)

Time profiles of the retronasal aroma compound *p*-cymene are shown in Fig. 2. The left figures show the raw data with a sampling rate of 0.1 s, and the right ones show the average for every 10 samples (every 1 s). All time profiles are shown in Figs. S2 (raw data) and S3 (averaged data). The results obtained when cumin on a spoon was held in the mouth (S-A–S-D) showed a tendency for the peak areas to gradually increase with time—suddenly and randomly increasing and then decreasing or remaining undetected for short periods (Fig. S3). Under these experimental conditions, the concentration of *p*-cymene in the space of the oral cavity gradually increased as the manner of breathing slightly and/or unconsciously changed with each breath.

As shown in Fig. S3, in the case of the results when eating only cumin, 0-A and 0-B showed intermittent appearances of the signal, whereas 0-C and 0-D showed only an occasional appearance. Powdered cumin was used in the present study, and this is not normally chewed. On the other hand, panelists performed the required chewing motion of the powdered cumin and swallowed it following 60 s in the present study. Therefore, panelists were able to consciously mix the powder with saliva to make a slurry to aid in swallowing. However, the movements of chewing and the tongue might not have been smoothly synchronized, and if the saliva and powder were not sufficiently mixed this could have resulted in irregular vaporization of the *p*-cymene in the oral cavity, which would have been followed by the random release of a retronasal aroma component. In addition, when eating only cumin, saliva gradually accumulated in the oral cavity, and panelists could have sometimes closed their throat to prevent saliva flow toward the throat during chewing.

In the case of eating the cumin sandwiches, the peak areas of *p*-cymene were roughly repeatedly increased and decreased in synchronization with the breathing cycle. However, the peak areas were not necessarily maximized just after placing the sample; that is, in many cases, the peak areas increased and then reached a maximum after a few breaths. These results suggest that since the cumin existed between the pieces of bread, it was unlikely to have been vaporized immediately after the chewing motion. Moreover, eating pieces of bread with cumin more closely approximated so-called usual eating conditions, which allowed panelists to chew smoothly and to unconsciouly synchronize the movement of chewing with the tongue. As a result, the overall signal behavior was smoothly attenuated after the initial increase.

The occurrence of two signal behaviors after swallowing was interesting; some peak areas continued to decrease (or disappeared) while, on the contrary, others slightly increased (for example, 0-A, 1-C, 3-C in Fig. S3). A plausible reason for the latter case could be that when the sample passed through the throat and was sent to the stomach, either the concentration of *p*-cymene around the space of the pass temporarily increased or the saliva containing cumin adhered to the mucous membrane, from which *p*-cymene was released.

### Fit function for a time profile and quantitative evaluation

In the present study we discussed a fit function representing the time profile for the occurrence of a retronasal aroma compound during chewing cumin sandwiched between two pieces of bread before swallowing. As previously mentioned, a signal behavior indicated a combination of the increase and decrease according to each breath, with an increase first and then a decrease. If the overall signal behavior indicates a monotonic decrease, this would suggest that an exponential decay fitting is a strong candidate. On the other hand, in the present study the peak area did not necessarily reach its maximum at the first exhalation after placing the sample in the mouth; the signal increase was observed over several breaths in some cases.

As an overall signal behavior of the increase and then decrease, we considered a fit function using a function we previously proposed [17] while performing the real-time monitoring of an oil-in-water (O/W) emulsion using REMPI-TOFMS; when creaming an O/W emulsion, the signals arising from the oil component indicated an increase and then a decrease—this was the same behavior obtained in the present study. In the previous study, this signal behavior was expressed via a fit function that was constructed by subtracting two logistic functions, which were monotonically increasing functions. In the present study, this fit function was modified, and a fit function was proposed whereby a trigonometric function was multiplied via the previous fit function to represent an increase and a decrease by each breath, as depicted in the following equation.

$$I=\left[\frac{{A}_{1}}{1+{\mathrm{e}}^{-{a}_{1}(t-{\gamma }_{1})}}-\frac{{A}_{2}}{1+{\mathrm{e}}^{-{a}_{2}(t-{\gamma }_{2})}}\right] \cdot \left[1+\mathrm{sin}\frac{2\uppi }{T}(t-{t}_{\mathrm{c}})\right]$$

**Fig. 3:**
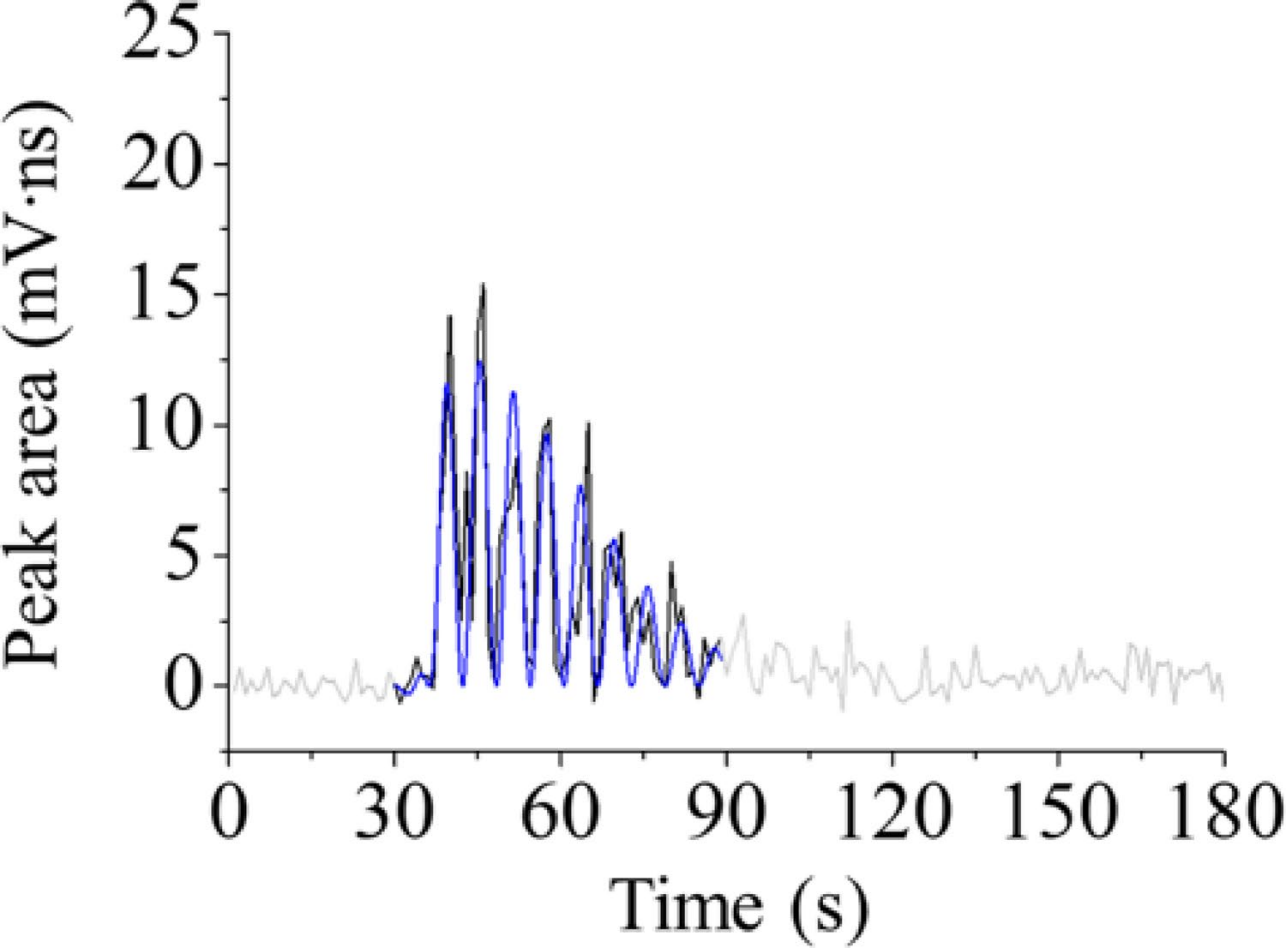
Fit result (blue line) for the time profile of the *p*-cymene of Fig. 2. The result of 7-A (1-s averaged) was fitted

This equation uses the subtracted terms of the logistic functions where *A* is the amplitude, *a* is the variable related to the slope at the inflection point (the slope is *aA*/4), *γ* is the time of the inflection point, and the subscripts 1 and 2 denote variables corresponding to each logistic function representing increasing and decreasing behaviors, respectively. In terms of the trigonometric function, *T* is a breathing cycle (realistically 6 s under the present experimental conditions) and *t*_c_ is the delay from the true time profile. The fit result of the averaged signal behavior of 7-A in Fig. 2 is shown in Fig. 3; all the fit results in Fig. S3 also appear in Fig. S4, and all the variables obtained in each fit are listed in Table 1. In the present study, the values of *A*_1_ and *A*_2_ were determined to be equal, and the values of *A* and *γ* were determined manually based on the outline of each experimentally obtained signal behavior. The other variables were calculated automatically.

**Table 1:** Calculated parameters in the fit equation for the time profiles shown in Fig. S4^α^

	Amount of bread [g]		
Parameter	1	3	7
$A_{{1}} \, \left[ {{\mathrm{mV}} \cdot {\mathrm{ns}}} \right]$	5.70	5.25	5.68
*a*_1_ [s^−1^]	0.465	2.14	1.62
$\gamma_1$ [s]	32.5	36.0	35.3
$A_{{2}} \, \left[ {{\mathrm{mV}} \cdot {\mathrm{ns}}} \right]$	5.70	5.25	5.68
*a*_2_ [s^−1^]	0.122	0.101	0.0858
$\gamma_{2}$ [s]	60.8	71.3	65.8
*t*_c_ [s]	1.96	1.19	0.899
*T* [s]	5.98	6.08	6.10

^α^ Means of parameters of four panelists

Compared with the signal behaviors obtained from the measurement with fitting results, occasionally the time profiles did not match; for example, very large peak areas appeared in the case of the experimental results, e.g., 1-A, 1-B and 7-D in Fig. S4. These results suggest that the concentrations of retronasal aroma components sometimes suddenly increase when a sandwich-type of food is eaten. Still, the above fit function roughly fits the experimental results; a breathing cycle, *T*, was calculated to 6 s in all fit results.

**Fig. 4:**
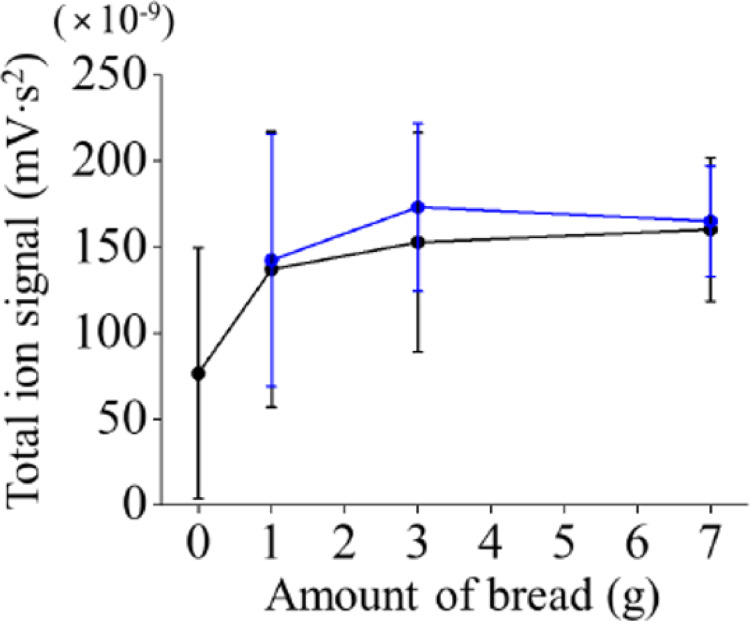
Relationship between the amount of bread and the total ion signal of *p*-cymene obtained during chewing. The total ion signals are the integrated areas from the experimentally obtained time profiles shown in Fig. S3 (black) and the fit results shown in Fig. S4 (blue). Error bars represent the standard deviation of the results obtained from four panelists

The relationship between the amount of bread and the total for the ion signals from *p*-cymene obtained during chewing (integrated area under the time profiles in Figs. S3 and S4) is shown in Fig. 4. The total of the ion signals obtained from both the experiments and fit results are shown, and these are roughly identical. In Fig. 4, the total of the ion signals from *p*-cymene obtained experimentally without bread, that is, with only cumin powder, is also included. First, all the experimental results showed large standard deviations. For example, the relative standard deviation (RSD) for the total of the ion signals obtained from the experiments when eating 3 g of bread was 41.8%. On the other hand, the results for the same measurements performed for one of the panelists showed both intraday and interday RSDs that were both much smaller (14.2% and 9.9%, respectively, n = 3). Therefore, the large standard deviation in Fig. 4 was attributed to individual differences rather than to poor reproducibility. Although there was no significant difference in the obtained values for each amount of bread, the total of the ion signals obtained when eating only cumin powder was about half that obtained with bread. As mentioned above, when cumin powder was placed on the tongue without bread, a signal for *p*-cymene was randomly detected. These results suggest that the volatilization of *p*-cymene without bread was suppressed because the powder cumin had mixed only with secreted saliva. On the other hand, in the cases of bread existing in the mouth, the powder cumin could have been spread throughout the oral cavity via the bread rather than mixing with saliva, which would have accelarated the volatilization of *p*-cymene. Incidentally, the total for ion signals obtained using 1 g of bread was slightly smaller than that obtained using the other amounts. Therefore, in the case of an even smaller amount of bread, the bread contained much saliva during a few breaths and became a liquid food tantamount to baby food, which could slightly decrease volatilization.

**Fig. 5:**
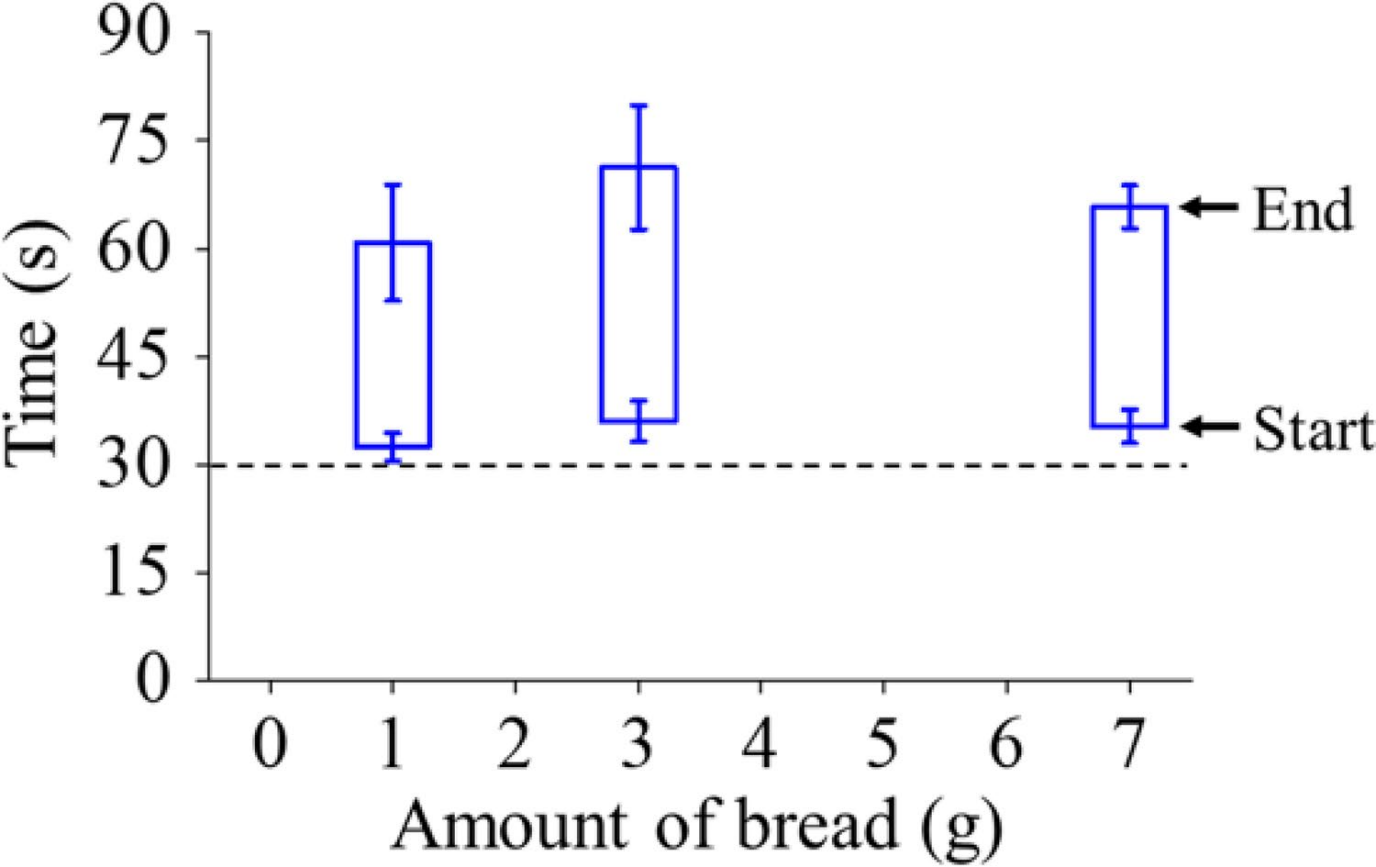
Relationship between the amount of bread and the start and end times and duration of the overall transient time profile of *p*-cymene as a retronasal aroma compound. The dashed line shows when the sample was placed into the oral cavity (30 s after starting the recording). Error bars represent the standard deviation of the results obtained from four panelists

The release time of a retronasal aroma component was also verified using the fit results of the inflection times of the start and end of the logistic functions (*γ*_1_ and *γ*_2_), as well as that of the duration time (*γ*_2_ – *γ*_1_) (Fig. 5). The start and end times also showed large variability. As an example, the standard deviations of both times when eating 3 g of bread were 2.8 s and 8.6 s, respectively. On the other hand, the standard deviations for the intraday of the same measurements for one panelist were 1.2 and 2.9 s, respectively (n = 3), and those of the interday were 2.1 and 2.6 s, respectively (n = 3). That is, the large standard deviations in Fig. 5, paticularly those of the end time, could probably be attributed to individual differences.

Likewise, there was no significant difference in the values of the start time, end time, and duration for each amount of bread. Therefore, in the following, the mean values were compared, and the causes of the observed differences were discussed only in terms of possible explanations. First, the start time, *γ*_1_, was slighly smaller with 1 g of bread than with the other amounts. These results indicate that *p*-cymene in cumin can quickly pass through the nose when the amount of bread is small. Quantitatively, the start time was approximately 3 s earlier, which is the time equivalent to half a breath when eating 1 g of bread as opposed to eating 3 or 7 g. Saliva was considered to have been scarcely secreted when bread samples were placed in the mouth, and the *p*-cymene in the cumin that was sandwiched between the slices of bread was vaporized earlier in the oral cavity with a smaller amount of bread. Moreover, the end time, *γ*_2_, was also smaller when eating 1 g of bread, and, as a result, the duration time, *γ*_2_ – *γ*_1_, was also short. This is probably because, when eating 1 g of bread, the pieces contained much saliva when chewed and became as a liquid food after a few breaths. As mentioned above, the total ion signal was slightly decreased when eating 1 g of bread (Fig. 4). However, as Table 1 shows, the values of amplitude, *A*_1_, were almost identical for any amount of bread; that is, the maximum concentrations of *p*-cymene, which were not a single instance but an average in the oral cavity, were almost the same. These results suggest that the decrease in the total ion signal when eating a smaller piece of bread could possibly heve been due to the decrease in the duration time rather than to a decrease in the maximum peak area.

Based on the obtained results, the correlation between eating conditions and the release behavior of the retronasal aroma was considered. When the amount of bread was small (1 g), the aroma components in cumin, such as *p*-cymene, were passed early from the oral cavity to the nose by easily loosening the sandwiching pieces of bread, but the duration became smaller because the proportion of saliva relative to the amount of bread became larger during chewing. Whether the amount of bread was 3 or 7 g, the release behavior seemed roughly the same, and, compared with the results obtained at 1 g, the start time of the aroma release was slowed by 3 s, which was equivalent to the time of half a breath in the present case, and the aroma persisted to some extent even while chewing. In any case, the obtained data exhibited considerable variability due to individual differences, and further verification will be necessary in future work.

Although the total ion signal could be calculated from the experimentally obtained time profile of the aroma components, it is difficult to quantitatively evaluate the start and end times along with the duration time because the release behavior of retronasal aroma components deviates extensively with each breath. However, using the proposed fit function, these times could be easily calculated. The present analytical method strongly contributes to an understanding of the volatilization behavior of aroma components in the oral cavity as well as to their release behavior toward the nose, which is useful for the design and evaluation of new foods that will enhance the enjoyment of aroma during eating.

## Conclusions

In the present study, a retronasal aroma compound was measured using REMPI-TOFMS in real time for the eating of cumin-sandwiched samples, and the correlation between the eating conditions and release behaviors was discussed. The time profile of *p*-cymene repeatedly increased and decreased with breaths. Moreover, a fit function was proposed for the obtained time profile, and, not only a total ion signal, but also the start and end times and duration could be quantitatively discussed. Although the obtained data show considerable variability due to individual differences and further verification is required, one possible explanation is that when eating a smaller sandwich of cumin, the cumin was exposed at an earlier stage of chewing, and the aroma compound,* p*-cymene, was released earlier into the oral cavity; however, relatively large amounts of saliva caused a rapid suppression of vaporization, and as a result the duration became shorter.

A nanosecond pulsed laser emitted at 266 nm was used in this study, which dictated that *p*-cymene would be the only detectable component from the cumin. In future work, we plan to investigate whether the parameters could be extracted in a more robust fashion under conditions where the sample is mixed with other spices or whether the parameters for other compounds could be extracted. Although cumin contains many other aroma compounds, the high level of selectivity from using REMPI-TOFMS allowed us to monitor the time profile of *p*-cymene without interference from all the other components. Of course, other compounds could be measured using a laser emitting at different wavelengths or a laser with a much shorter pulse width, such as a femtosecond laser [18], and organic compounds such as acetone have been measured from exhaled breath [10]. Evaluation of the release behavior of retronasal aroma compounds that is based on objective data should be beneficial for the development of foods more suited to consumer preferences as well as for proposals that could enhance the dining experience.

## Supplementary Information

Below is the link to the electronic supplementary material.

Supplementary file1 (PDF 333 kb)

## Data Availability

The datasets generated and/or analyzed during the current study are available from the corresponding author upon reasonable request.
