# Supplementary material for: Resonance-enhanced multiphoton ionization time-of-flight mass spectrometry for the real-time analysis of a retronasal aroma compound from a cumin sandwich
Source: Anal Sci. 2026 Apr 3;42(6):411–9. doi: 10.1007/s44211-026-00907-z (PMC13201309; doi:10.1007/s44211-026-00907-z)
Supplement: Supplementary file 1 — Supplementary file1 (PDF 333 kb) [file 44211_2026_907_MOESM1_ESM.pdf]

Resonance-enhanced multiphoton ionization time-of-flight mass spectrometry for the real-time analysis of a retronasal aroma compound from a cumin sandwich

<Supporting Information>

Hazuki Uno, Masaaki Ukita, and Tomohiro Uchimura†

Department of Materials Science and Engineering, Graduate School of Engineering, University of Fukui, 3-9-1 Bunkyo, Fukui 910-8507, Japan

† To whom correspondence should be addressed.

E-mail: uchimura@u-fukui.ac.jp

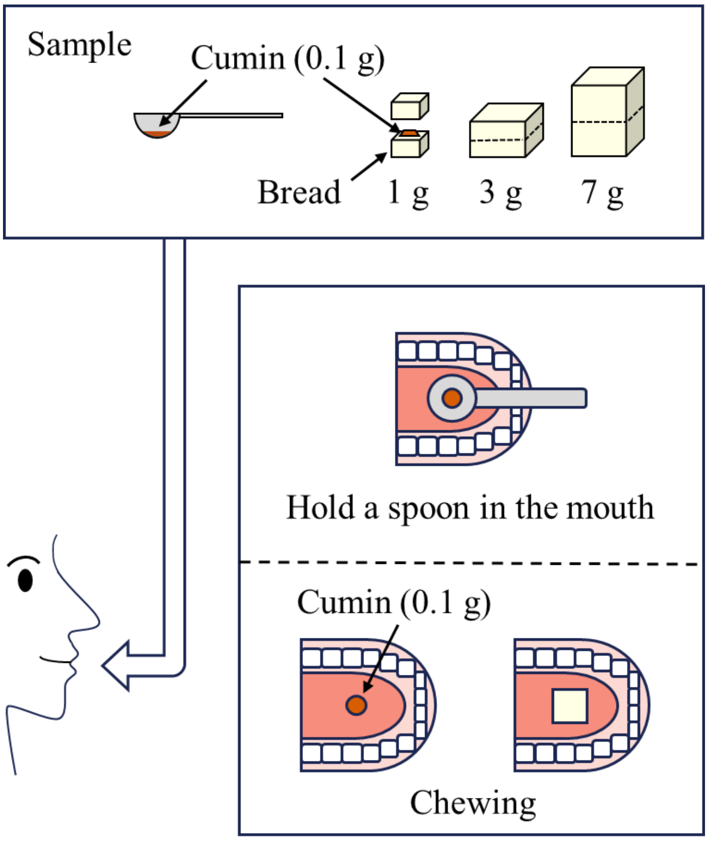

Fig. S1 Schematic of the food samples and their positions in the oral cavity.

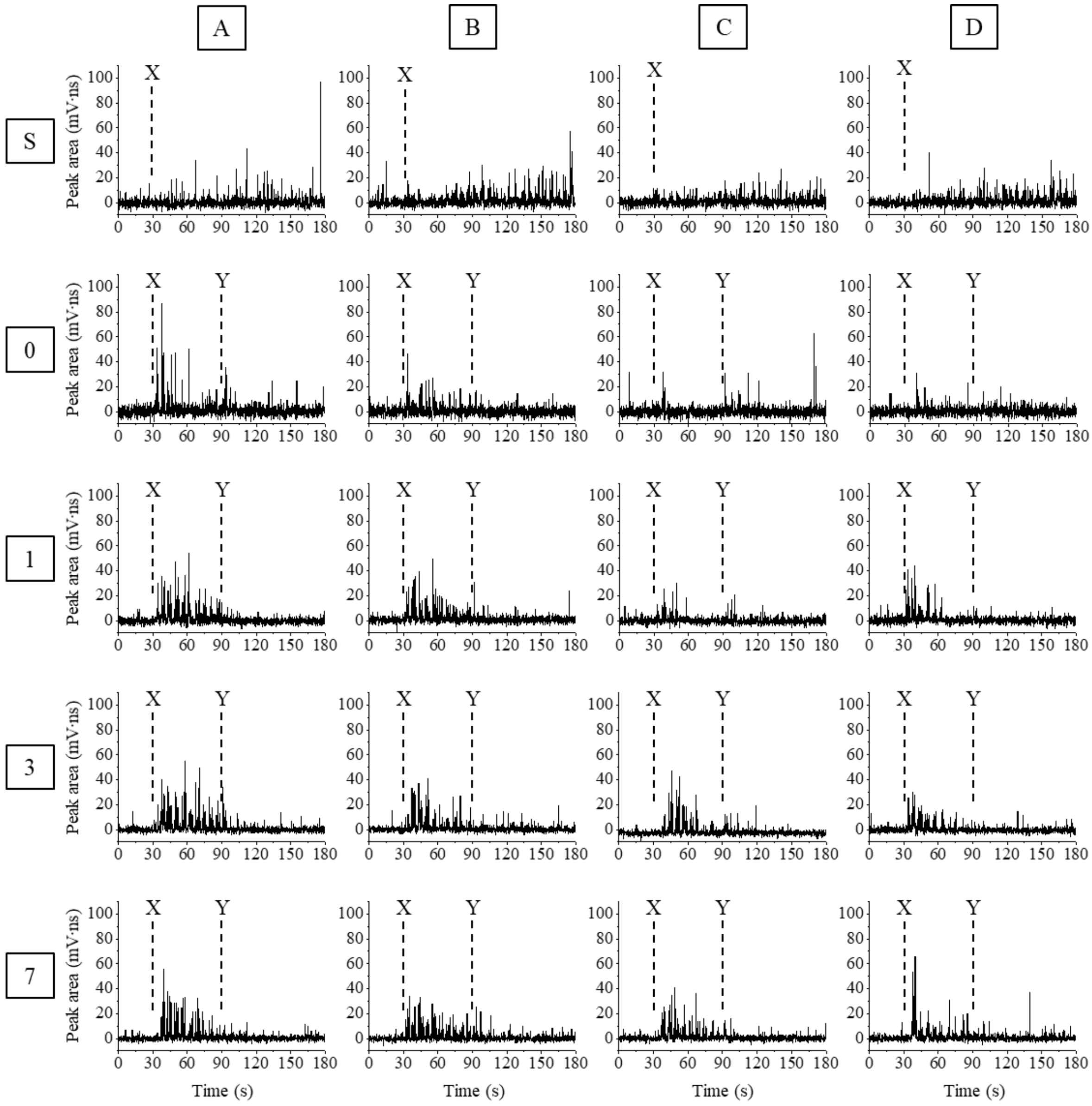

Fig. S2 All time profiles of *p*-cymene as a retronasal aroma compound obtained in the present study. S refers to the experiment where a spoon was placed in a mouth. The numbers 0 to 7 indicate the total amount (g) of two pieces of bread (0 means that only cumin was placed on the tongue). A-D refers to a respective panelist. X indicates when the sample was placed into the oral cavity (30 s after starting the recording). Y indicates when the panelist swallowed the sample (60 s after starting the eating).

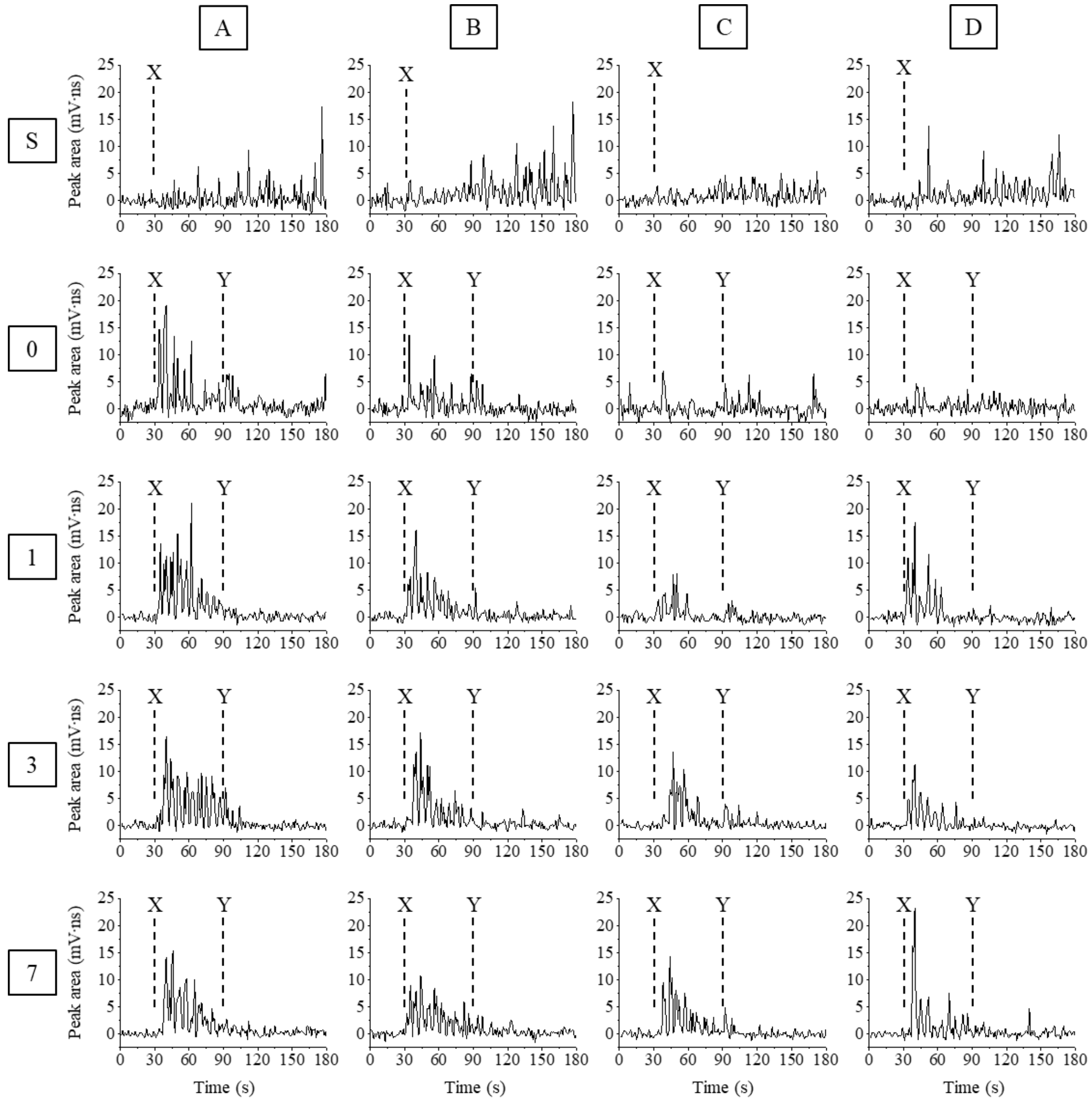

Fig. S3 Averaged time profiles of Fig. S2 for every 1 s. S refers to the experiment where a spoon was placed in a mouth. The numbers 0 to 7 indicate the total amount (g) of two pieces of bread (0 means that only cumin was placed on the tongue). A-D refers to a respective panelist. X indicates when the sample was placed into the oral cavity (30 s after starting the recording). Y indicates when the panelist swallowed the sample (60 s after starting the eating).

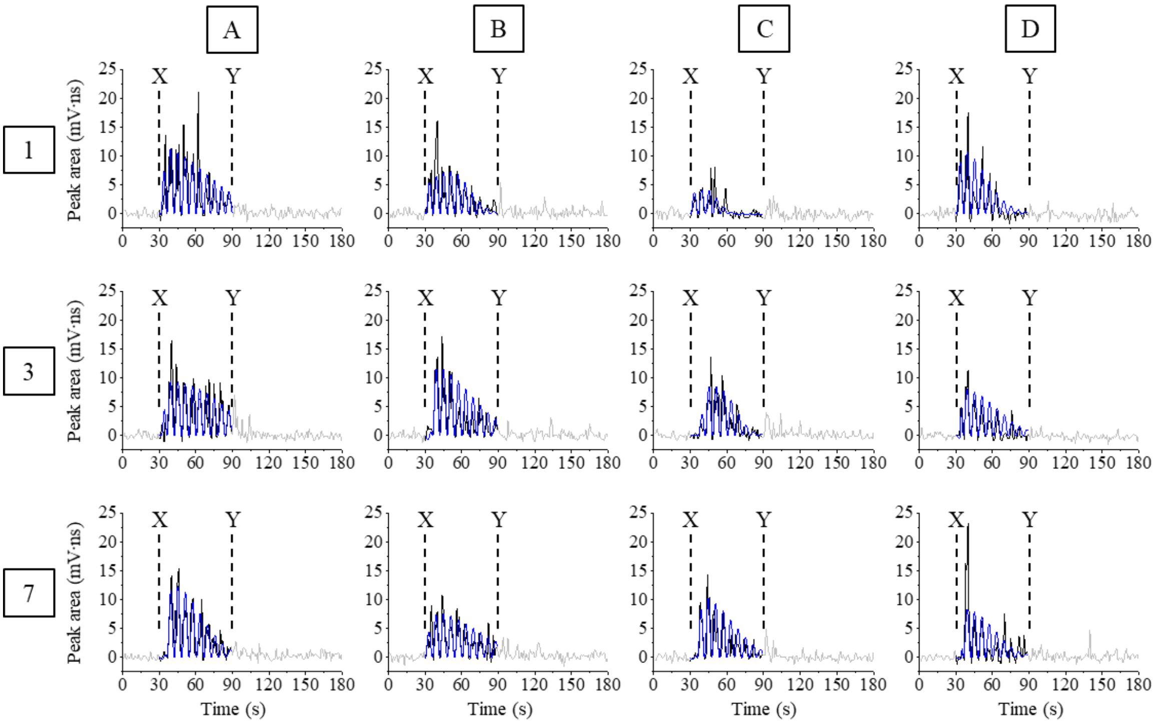

Fig. S4 Fit results (blue line) for the time profiles of *p*-cymene obtained when eating 1, 3 and 7 g of two pieces of berad in Fig. S3.
